# Simultaneous Foxp3 and IDO expression is associated with sentinel lymph node metastases in breast cancer

**DOI:** 10.1186/1471-2407-9-231

**Published:** 2009-07-15

**Authors:** Aaron S Mansfield, Paivi S Heikkila, Ari T Vaara, Karl AJ von Smitten, Jukka M Vakkila, Marjut HK Leidenius

**Affiliations:** 1 Breast Surgery Unit, Helsinki University Central Hospital, Helsinki, Finland; 2 Department of Pathology, Helsinki University Central Hospital, Helsinki, Finland; 3 Haematology Research Unit, Helsinki University Central Hospital, Finland

## Abstract

**Background:**

There is evidence that the immune systems of patients with breast cancer are dysfunctional. Regulatory T cells (Tregs), and IDO, an immunosuppressive enzyme, are associated with more advanced disease in some cancers and may promote immunologic tolerance to tumors. Our aim was to assess whether expression of Foxp3, a marker of Tregs, and IDO were linked with nodal metastasis in breast cancer patients. Inhibitors of IDO are available and could potentially demonstrate utility in breast cancer if IDO drives progression of disease.

**Methods:**

Sentinel lymph nodes (SLN) of 47 breast cancer patients with varying degrees of nodal disease and 10 controls were evaluated for expression of Foxp3 and IDO using immunohistochemistry. Positively stained cells were quantified and their distribution within the SLN noted.

**Results:**

The proportion of Foxp3^+ ^cells was higher in SLN of cancer patients than controls (19% v. 10%, p < 0.001). Specifically, there were more Foxp3^+ ^cells in SLN with metastasis than tumor-free SLN (20% v. 14%, p = 0.02). The proportion IDO^+ ^cell in SLN of cancer patients was not statistically different than controls (4.0% v. 1.6%, p = 0.08). In order to demonstrate the combined immunosuppressive effect of Foxp3 and IDO, we categorized each SLN as positive or negative for Foxp3 and IDO. The Foxp3^+^/IDO^+ ^group almost exclusively consisted of cancer patients with node positive disease.

**Conclusion:**

In conclusion, our study shows that Foxp3^+ ^cells are associated with more advanced disease in breast cancer, a finding that is proving to be true in many other cancers. As IDO has been found to promote differentiation of Tregs, IDO may become a suitable target to abrogate the development of T-cell tolerance and to promote an effective immune response to breast cancer. Our results about the combined expression of IDO and Foxp3 in metastastic SLN support this assumption.

## Background

By the time cancer is clinically detectable tumors have likely developed mechanisms to escape immunosurveillance, and increasing evidence suggests that regulatory T-cells (Tregs) have a major role in modulating host response to tumor. Tregs are produced in the thymus and they act in the periphery to control potentially hazardous self-reactive effector cells that have escaped thymic negative selection[1]. The exact mechanism of suppression is not yet determined; however, direct cellular contact is required. Regardless, it has been shown that accumulation of Tregs in tumor, as measured by Foxp3 expression, has been associated with a worse prognosis in patients with ovarian cancer[2,3]. In esophageal and gastric cancers higher populations of tumor infiltrating Tregs were associated with more advance disease[4]. Further evidence for the role of Tregs in tumor-specific immunosuppression is seen in murine models where depletion of Tregs promotes an effective antitumor response[5].

Indolamine 2,3-dioxygenase (IDO) is a tryptophan degrading-enzyme that inhibits T-cell proliferation. IDO is expressed by trophoblasts, dendritic cells[6] and macrophages[7]. IDO has been found to prevent fetal rejection[8], and it is suspected that it may promote tolerance to tumors[9]. There is evidence that there is increased expression of IDO in the primary tumor and serum of patients with breast cancer[10], and that higher levels of IDO expression in colorectal carcinoma represent a poor prognostic factor[11]. Additionally, expression of IDO by leukemic cells in acute myeloid leukemia has been shown to induce Foxp3^+ ^Tregs[12]. The role of IDO has been considered relevant enough that inhibitors of IDO are being developed for clinical trials[13]. These inhibitors could potentially demonstrate utility in breast cancer if IDO promotes progression of disease.

Metastasis to lymph nodes is one of the strongest predictors of survival in patients with breast cancer [14-16]. Axillary clearance for lymph node evaluation has been replaced by sentinel lymph node biopsy at many centers in the setting of the appropriate clinical stage[17]. Improved selectivity in lymph node dissection has resulted in a more detailed analysis of the removed nodes, and greater attention is being paid to the immune status of these nodes. Sentinel lymph nodes (SLN) are unique in that they represent both the sites of T-cell activation and early metastasis. As such, these nodes are ideal for investigations of tumor immunology in breast cancer.

Recent reports have shown that the immune systems of patients with breast cancer are dysfunctional[18]. Therefore, we set out to find evidence of immunosuppression in the SLN of patients with breast cancer. We hypothesized that the expression of Foxp3 and IDO within SLN were associated with nodal metastasis.

## Methods

We used SLN of 47 breast cancer patients treated at the Breast Surgery Unit of Helsinki University Hospital between 2001 and 2003. A prospectively collected database was searched to select patients with node negative disease as well as micro- and macro-metastasis. Another criterion was to include patients that also had non-SLN harvested. These patients had either tumor negative SLN (n = 11), SLN with micrometastases (n = 16), or SLN with macrometastases (n = 20) as defined by the International Union Against Cancer (UICC) TNM classification system[19]. The clinicopathological data of the breast cancer patients included in this study are summarized in Table 1. SLN from 10 control patients with SLN biopsy due to a false-positive preoperative biopsy, either core needle biopsy or fine needle aspiration cytology, were used as controls. These control patients had premalignant conditions such as lobular neoplasia in situ or atypical ductal hyperplasia, but not ductal carcinoma in situ or invasive breast cancer. This project was approved by the Ethics Committee of Helsinki University Central Hospital.

**Table 1:** Patient and Tumor Characteristics

	Node-positive	Node-negative
**Number**	36	11
Age*	60 (49–70)	54 (48–70)
**Type**		
Ductal	20	5
Lobular	15	3
Other	2	3
Tumor Size (mm)*	16 (14–23)	17 (14–22)
**Tumor Stage**		
T1b	5	1
T1c	22	6
T2	12	5
**Tumor Grade**		
I	8	4
II	24	3
III	4	4
**ER**		
Positive	34	9
Negative	2	2
**PR**		
Positive	23	8
Negative	13	3
**MIB-1**		
Positive	15	5
Negative	21	6

There were no statistically significant differences between the groups.

ER = estrogen receptor, PR = progesterone receptor, MIB-1 = proliferation index.

* median (range)

The SLN were assessed using serial sectioning and immunohistochemistry[20,21]. One representative tissue block was selected for immunohistochemical studies upon evaluation of all available material.

### Immunohistochemistry (IHC)

Formalin-fixed, paraffin-embedded lymph nodes were sectioned into 3–4 μm slices and affixed on glass slides. After the samples were heated for half an hour at 56°C, they were deparaffinized in xylene, rehydrated in a graded alcohol series, and washed in water. For antigen retrieval, samples were microwaved for a total of 20 minutes in a citrate buffer (pH = 6). Endogenous peroxidase activity was quenched in a bath of methanol and hydrogen peroxide for 30 minutes. Samples were incubated overnight at 4°Celsius with Foxp3 antibodies (Nordic Biosite AB Täby, Sweden) and IDO antibodies (Millipore Oy Espoo, Finland) at concentrations of 1:1000 and 1:100, respectively. These antibodies were detected with Vectastain Elite ABC Rat IgG kit (Vector Burlingame, CA). Samples were incubated overnight at 4°Celsius with CD3 (Dako, Denmark) and CD4 (Novocastra, Great Britain) antibodies at concentrations of 1:300 and 1:25, respectively, and were detected with Envision Advance-Kit (Dako, Denmark). All samples were counterstained with Harris Hemotoxylin for 30 seconds and mounted. Samples were run in batches with as many as 19 at a time. Batches contained a mixture of controls, node negatives and node positives.

Positively stained cells in the non-metastatic regions of the lymph nodes were quantified as a percentage of all cells in a minimum of three high-powered fields (400× magnification), and averaged. In some cases, fewer fields were able to account for all of the positive staining. Non-metastatic regions were used to allow comparison of lymphocytes between patients with and without metastasis. A Nikon eclipse 80i microscope was used for data analysis and image acquisition.

### Statistics

JMP (SAS Institute Inc., USA) was used for statistical analysis. Basic statistics were used to analyze our grouped data. Since the data are non-parametric, the Kruskal-Wallis and the Mann-Whitney U tests were used to compare the expression of each stain amongst the groups. For the stains with statistically significant differences in expression, multiple pairwise comparisons were made using Wilcoxon's rank sum test. Spearman's rank-order test was used to assess for correlations between each stain and clinicopathologic data. Logistic regression analyses were performed to assess the predictive effect each stain had on nodal status.

The Foxp3 and IDO stainings were divided into negative and positive groups according to their medians. These cutoffs were used to form four nodal-suppression profiles: Foxp3^+^/IDO^+^, Foxp3^+^/IDO^-^, Foxp3^-^/IDO^+^, and Foxp3^-^/IDO^-^. The proportions of patients in these four categories of SLN were then compared between the node-positive and the node-negative cancer patients and the controls using χ^2 ^statistical test.

## Results

### Foxp3^+ ^lymphocytes are located in the paracortex of SLN

**Figure 1:**
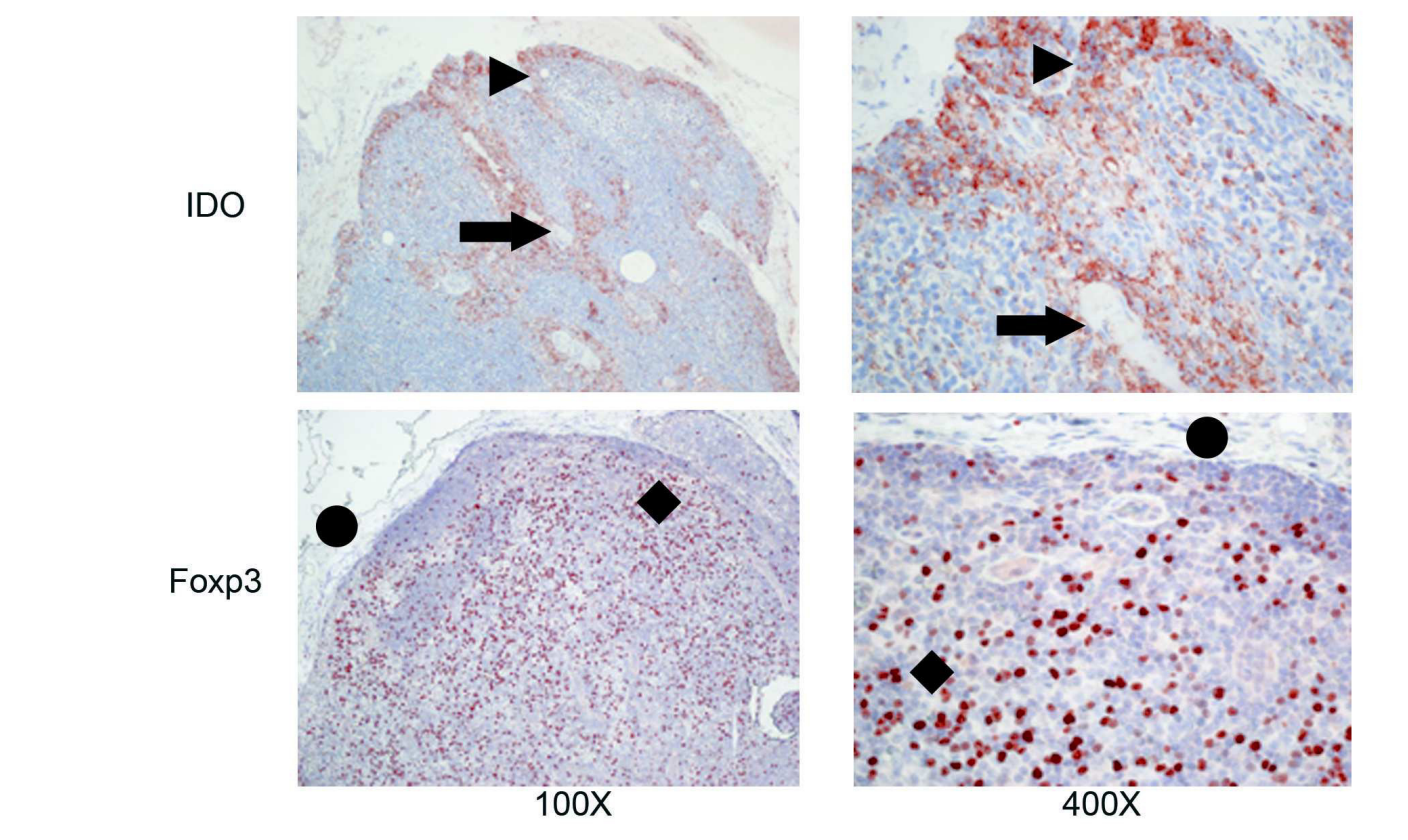
**Foxp3 and IDO expression in the sentinel lymph nodes (SLN)**. This figure illustrates the expression of IDO (top row) and Foxp3 (bottom row) in a SLN with a 7 mm breast cancer metastasis at 100× (first column), and 400× (second column) magnification. Foxp3^+ ^cells are located primarily in the paracortical regions, whereas IDO^+ ^cells infiltrate perisinusoidal areas. The pericapsular fat is marked by the circle, the paracortex by the diamond, the marginal sinus by the arrowhead, and the medullary sinus by the diamond.

Foxp3^+ ^cells were morphologically small lymphocytes and were distributed in the typical T-cell areas, namely the paracortical regions of SLN, Figure 1. The localization was similar in SLN from the cancer patients and the controls.

### Metastatic SLN of cancer patients are enriched with Foxp3^+ ^cells

**Figure 2:**
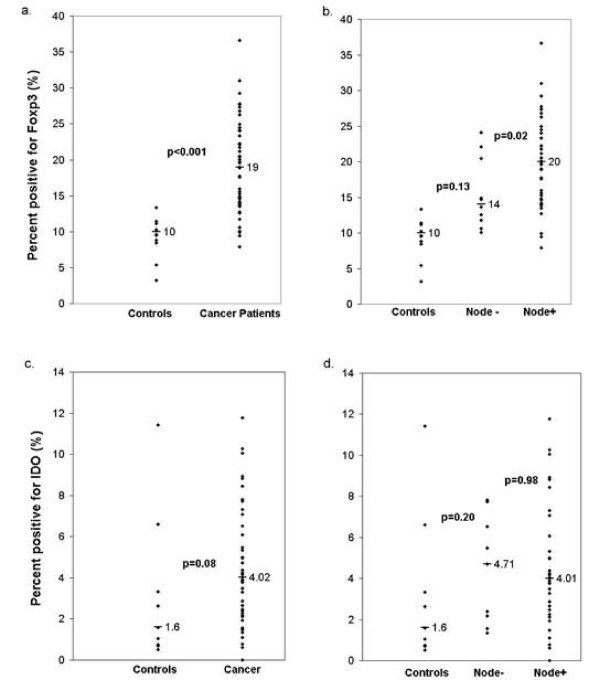
**Foxp3 and IDO expression in the sentinel lymph nodes of breast cancer patients and controls**. The expression of Foxp3 is shown as the median percentage of positive cells in the sentinel lymph nodes of breast cancer patients and controls (a), and of node-positive, node-negative breast cancer patients and controls (b). The expression of IDO is shown as the median percentage of positive cells in the sentinel lymph nodes of breast cancer patients and controls (c), and of node-positive, node-negative breast cancer patients and controls (d).

The relative frequency of Foxp3^+ ^cells was higher in cancer patients, median 19%, range 8–37%, than in controls, median 10%, range 3–13%, p < 0.001, Figure 2a. Cancer patients were further divided into two subgroups: those with metastases to SLN and those with tumor-free SLN. A higher proportion of Foxp3^+ ^cells were observed in metastatic SLN, median 20%, range 8–37%, than in SLN without metastasis, median14%, range 8–24%, p = 0.02, Figure 2b. No difference in the expression of Foxp3^+ ^was observed between SLN with micro- and macrometastases, p = 0.64. The proportions of Foxp3^+ ^cells in SLN of node-negative patients and controls were not significantly different from each other, p = 0.13. Foxp3 expression was a significant predictor of nodal status, p = 0.001.

The higher proportion of Foxp3^+ ^cells in the SLN of the cancer patients was neither due to increased infiltration of CD3^+ ^T-cells nor CD4^+ ^T-cells. The ratio of Foxp3^+ ^cells to CD3^+ ^cells was higher, 0.24, among the cancer patients when compared with the ratio of 0.14 in the controls, p < 0.001. Similarly, the ratio of Foxp3^+ ^cells to CD4^+ ^cells was higher, 0.29, in the cancer patients than in the controls, 0.14, p < 0.001. The ratios of Foxp3^+^:CD4^+ ^and Foxp3^+^:CD3^+ ^were greater in SLN with metastases compared to tumor-free SLN, p < 0.001, Table 2.

**Table 2:** The mean Foxp3/CD3 and Foxp3/CD4 ratios according to the patient group

	Cancer	Node positive	Node negative	Control
Foxp3:CD3*	0.24	0.26	0.17	0.14
Foxp3:CD4*	0.29	0.31	0.19	0.14

Ratio of Foxp3^+ ^cells to that of other T cell markers.

* p < 0.001 for cancer patients compared to controls, and node-positive patients compared to node-negative patients.

### IDO^+ ^cells are macrophages located in the perisinusoidal areas of SLN

The IDO^+ ^cells were located mainly in the perisinusoidal areas of the SLN, both around marginal and medullary sinuses. Most IDO^+ ^cells had the morphologic appearance of macrophages, Figure 1. IDO^+ ^cells that had a morphologic appearance typical of dendritic cells were only occasionally observed in the paracortical regions of SLN. The localization of IDO^+ ^cells was similar between SLN from cancer patients and controls.

### IDO expression is not associated with SLN status in breast cancer

In accordance with Foxp3 expression, a higher frequency of IDO^+ ^cells was observed in SLN of breast cancer patients (median 4.0%, range 2.3–6.0%) when compared to controls (median 1.6%, range 0.8–3.3%), but the difference was not statistically significant, p = 0.08, Figure 2c. The median proportion of IDO^+ ^cells in SLN with metastases was 4.0% (range 2.5–6.0%) and the proportion without metastases was 4.7% (range 2.2–6.5%), p = 0.98, Figure 2d. IDO expression was not a significant predictor of nodal status using a logistic regression model, p = 0.42.

### Expression of Foxp3 and IDO is correlated in tumor-free SLN

**Figure 3:**
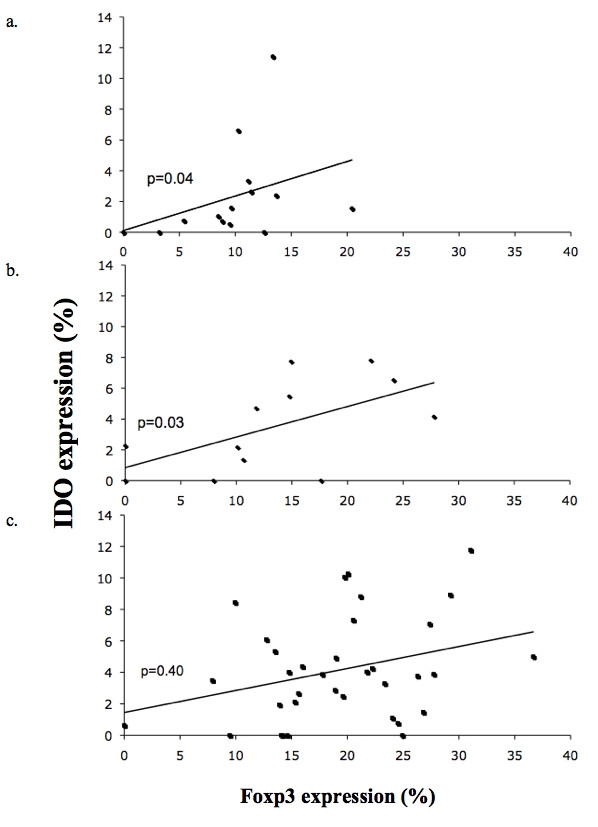
**Correlation of the infiltration of Foxp3^+ ^and IDO^+ ^cells**. The correlation of Foxp3 and IDO expression of controls (a), node-negative (b), and node-positive (c) patients is graphed above. There were significant correlations in the expression of both markers between the sentinel lymph nodes of the controls (p = 0.04) and those of patients with tumor-free sentinel lymph nodes (p = 0.003).

The expression of Foxp3 was positively correlated with that of IDO in SLN of controls, Spearman's rank coefficient r = 0.69, p = 0.04, as well as in SLN without nodal metastasis, r = 0.66, p = 0.003. In tumor-positive SLN no correlation was found, r = 0.16, p = 0.4, Figure 3.

### Simultaneous Foxp3 and IDO expression is associated with SLN metastases

**Figure 4:**
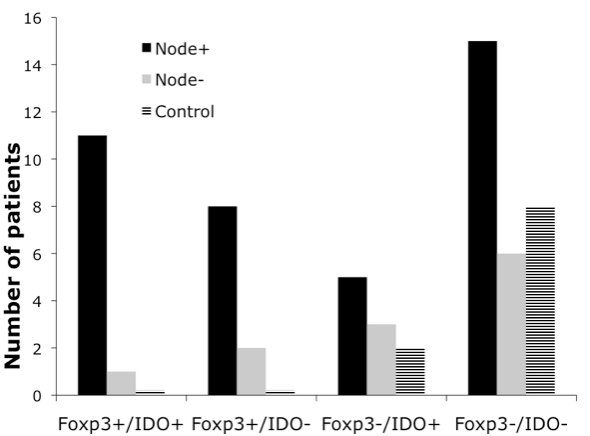
**Simultaneous Foxp3 and IDO expression is associated with sentinel lymph node metastases in breast cancer**. In order to demonstrate the combined immunosuppressive effect of Foxp3 and IDO, we categorized each subject into one of four groups based on whether positive or negative for Foxp3 and IDO. The median proportion of Foxp3^+ ^and IDO^+ ^cells in the SLN in cancer patients were used the cut-off points. Subjects who were positive both for Foxp3 and IDO were almost exclusively those with nodal disease, (p = 0.007).

In order to demonstrate the combined immunosuppressive effect of Foxp3 and IDO, we categorized each SLN into one of four groups based on whether the SLN was positive or negative for Foxp3 and IDO. The median proportion of Foxp3^+ ^cells and IDO^+ ^cells in the SLN of the cancer patients were used as cut-off levels. Values above 19% for Foxp3, and above 4% for IDO were considered positive. The Foxp3^+^/IDO^+ ^group consisted almost exclusively of SLN with metastatic disease, whereas the Foxp3^-^/IDO^- ^group contained almost all of the control SLN (p = 0.007, Figure 4). Nominal logistic regression analysis however did not show that IDO improved the predictive effect of Foxp3 on nodal status.

## Discussion

We studied SLN in breast cancer patients and observed an accumulation of Foxp3^+ ^cells in metastatic lymph nodes. Previous studies have pointed out that tumor infiltrating Foxp3^+ ^Tregs may influence the clinical course of breast cancer. Bates and co-workers[22] quantified Foxp3^+ ^Tregs in primary ductal carcinoma in situ (DCIS) and invasive breast cancer and found that higher numbers of Foxp3^+ ^Tregs in patients with DCIS correlated with increased risk of relapse. Furthermore, in patients with invasive breast cancers, infiltration of the primary tumors with Tregs was associated with lymph node involvement and increased risk of relapse after five years. Interestingly, a recent study by Ladoire[23] demonstrated that clinical response to neoadjuvant therapy with an anthracycline-based regimen was associated with a clearance of Foxp3^+ ^cells from primary tumors. This finding indicates that the immune system may be involved in eradication of cancer cells in the context of cytotoxic chemotherapy.

Our results demonstrating Foxp3^+ ^cell infiltration within metastatic SLN of breast cancer patients are in line with other studies that correlate high numbers of Tregs with a metastatic and invasive growth pattern of cancer. In addition to breast cancer, Tregs may have a role in many other cancers. In metastatic lymph nodes of patients with melanoma there are higher frequencies of Tregs than in tumor-free lymph nodes[24]. In contrast, in ovarian cancer fewer Tregs were found in lymph nodes from patients with advanced stage disease than in earlier stages; however, accumulation in tumor and ascites was correlated with more advance stage[2]. In our patient population an association between accumulation of Foxp3^+ ^cells in SLN and distant recurrence (data not shown) was not observed. This may not be surprising, since most of our patients had tumors with favorable prognostic factors such that the number of relapses has remained low. Also, follow-up time was too short to evaluate recurrences after five years. Our findings are also supported by Matsuura[25] who used quantitative real-time reverse transcriptase-polymer chain reaction to find significantly higher levels of expression of Foxp3 mRNA in SLN with metastasis than in those without.

It has recently been shown that IDO expression by plasmacytoid dendritic cells promotes differentiation of Foxp3^+ ^Tregs[26]. Furthermore, the expression of IDO has been correlated with an increase of Foxp3^+ ^cells in multiple murine models [27-29], in human pancreatic adenocarcinoma[29], in human uterine cervical carcinoma[30], and in humans with advanced stages of melanoma following vaccination[31]. Even though few IDO^+ ^cells were found in the Foxp3^+^-rich paracortical regions of SLN, IDO depletes tryptophan and may have regional effect. Reduced levels of tryptophan activate the amino acid sensitive GCN2-kinase pathway and result in activation of Tregs[32]. Accordingly, cell-to-cell contact may not be necessary for IDO^+ ^cells to exert their effect. It is uncertain if the upregulation of Tregs may be strictly due to IDO, or tumor or if another mechanism exists.

Based on these reports, we studied the proportion of IDO^+ ^cells in SLN of our patients. Similar to Foxp3 expression, SLN from breast cancer patients contained higher numbers of IDO producing cells; however, the difference between cancer and control patients was not statistically significant, and IDO expression did not predict nodal status in a regression analysis. In tumor-free SLN of breast cancer patients and in controls a correlation between Foxp3^+ ^and IDO^+ ^cells was observed. In contrast, no correlation was found in metastatic lymph nodes suggesting that the infiltration of breast cancer cells into SLN interferes with the dependence of IDO and Foxp3 on each other. The expression of IDO may attract or induce Foxp3^+ ^cells prior to nodal metastasis. Localization of IDO^+ ^cells was not influenced by tumor cell invasion as IDO^+ ^cells were found in perisinusoidal areas in nonmetastatic and metastatic SLN. In melanoma patients IDO expression in SLN has been linked with the immunosuppressive cytokine IL-10. In these SLN the expression of IDO and IL-10 was found to be highest in SLN that were infiltrated with melanoma cells[33]. Another study also found greater levels of expression of IL-10, IFN-γ, and IDO in SLN with metastasis than non-SLN in patients with melanoma[34].

Concurrent expression of IDO and Foxp3 in SLN correlated well with lymph node metastasis. Only 1 out of 12 patients that had high numbers of Foxp3^+ ^and IDO^+ ^cells in SLN had node negative disease. Furthermore, most cases of node negative disease and almost all of the controls were in the group with low numbers of Foxp3^+ ^and IDO^+ ^cells. This finding suggests that IDO may be involved with the induction of T-cell tolerance in these patients. However, regression analysis did not demonstrate a combined predictive effect of Foxp3 and IDO on nodal status. This indicates that the effect of IDO may be masked by the strong influence of Foxp3^+ ^cells in the present material.

In this study, immunohistochemistry was used for analysis. Although IHC may be less sensitive and more subjective in the interpretation than PCR, it allows lymph node structure to be taken into consideration during analysis. Due to the use of IHC, we were able to analyze non-metastatic regions of SLN whereas PCR does not distinguish between expression by tumors and immunocytes. The morphological appearance and distribution of Foxp3^+ ^as well as IDO^+ ^cells in the lymph node attests to the quality and reliability of the staining. We found that Foxp3^+ ^cells were small lymphocytes located in the typical T-cell areas, while the IDO^+ ^cells were morphologically macrophages located at perisinusoidal areas.

## Conclusion

In conclusion, our study shows that Foxp3^+ ^cells are associated with more advanced disease in breast cancer, a finding that is proving to be true in many other cancers. As IDO has been found to promote differentiation of Tregs, IDO may become a suitable target to abrogate the development of T-cell tolerance and to promote an effective immune response to breast cancer. Our results about the combined expression of IDO and Foxp3 in metastastic SLN support this assumption.

## Abbreviations

SLN: sentinel lymph nodes; Tregs: regulatory T-cells; IDO: indolamine 2,3-dioxygenase; DCIS: ductal carcinoma in situ; ER: estrogen receptor; PR: progesterone receptor; MIB-1: proliferation index.

## Competing interests

The authors declare that they have no competing interests.

## Authors' contributions

AM, JV, PH, KvS, and ML participated in the concept and design of the study. AM, AV, and ML participated in data acquisition. AM, JV, PH, and ML participated in data analysis, interpretation of data and drafting of the manuscript. All authors read and approved the final manuscript.

## Pre-publication history

The pre-publication history for this paper can be accessed here:

http://www.biomedcentral.com/1471-2407/9/231/prepub
